# ATP Competitive Protein Kinase C Inhibitors Demonstrate Distinct State-Dependent Inhibition

**DOI:** 10.1371/journal.pone.0026338

**Published:** 2011-10-17

**Authors:** Ida M. Smith, Naoto Hoshi

**Affiliations:** Department of Pharmacology, University of California Irvine, Irvine, California, United States of America; Cardiff University, United Kingdom

## Abstract

We previously reported that some ATP competitive protein kinase C (PKC) inhibitors are either competitive or uncompetitive inhibitors with respect to substrate peptides. In this report, we demonstrate how the interactions between PKC and inhibitors change PKC activation kinetics. A substrate competitive inhibitor, bisindolylmaleimide I, targets activated PKC and stabilizes PKC in the activated conformation. This leads to transient activation and prolonged deactivation of PKC in the presence of bisindolylmaleimide I. In contrast, an uncompetitive substrate inhibitor, bisindolylmaleimide IV, targets quiescent PKC and stabilizes PKC in the quiescent conformation, which generates slower activation and suppressed translocation upon activation of PKC.

## Introduction

ATP competitive kinase inhibitors have been widely used to identify signaling pathways. In some cases, however, pharmacological observations do not support the biochemical data. One example is the acetylcholine induced suppression of the M-type potassium channel [1], [2] It has been known that this regulation involves protein kinase C (PKC) activation [3], [4], [5]. However, some PKC inhibitors do not prevent the suppression of the M-current induced by muscarinic agonists, which once led to an exclusion of PKC from the list of candidate mediators [2], [6], [7]. We found that this discrepancy is due to a PKC associating protein, AKAP79/150, which tethers PKC in the M-channel complex [4]. We demonstrated that AKAP79/150 bound PKC cannot interact with some PKC inhibitors, such as bisindolylmaleimide I (BIS I), since the pseudosubstrate-like domain in the PKC binding domain of AKAP79/150 competes with BIS I binding [8]. Through this study, we identified BIS I as a competitive inhibitor with respect to substrate peptides. In addition, we found that a related molecule, BIS IV, is an uncompetitive inhibitor for the substrate peptide. These results suggest that ATP competitive PKC inhibitors can modify how PKC interacts with substrate peptides.

Potential interactions between substrate peptides and ATP competitors are also suggested by crystal structure studies. To date, several crystal structures of PKC-inhibitor complexes have been solved [9], [10], [11], [12]. These analyses demonstrated that such ATP competitor molecules make hydrogen bonds with residues located in the substrate recognition groove. Thus, the structural information is consistent with a hypothesis that some PKC inhibitors compete not only with ATP but also with substrate peptides or pseudosubstrates. However, how ATP competitive kinase inhibitors interact with the pseudosubstrate domain remains unknown.

The pseudosubstrate domain governs the activation status of many serine/threonine kinases [13]. PKC is a typical example of such kinases [14], [15]. In the quiescent state, the pseudosubstrate covers the catalytic site so that no substrate proteins can be phosphorylated. Upon activation, a conformational change uncovers the catalytic site from the pseudosubstrate domain. This allows substrate proteins to enter the catalytic site for phosphorylation.

In this paper, we investigate functional consequences of the interaction between the intramolecular pseudosubstrate domain of PKC and ATP competitive inhibitors. We show that the primary target for BIS I is activated PKC while BIS IV targets quiescent PKC. We demonstrate that these different state-dependent inhibitions change the activation kinetics of PKC and stabilize PKC in certain conformations within the cellular environment.

## Results

### Time-dependent changes in potencies of BIS compounds

**Figure 1:**
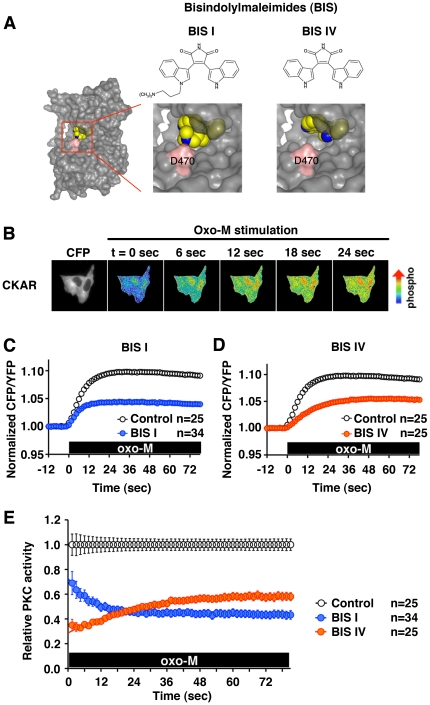
Two BIS compounds show distinct time-dependent changes in potency affecting cellular PKC activities. (**A**) Molecular structure of bisindolylmaleimide I (BIS I, left) and bisindolylmaleimide IV (BIS IV, right). Lower panels show molecular models for BIS bound PKCβII modified from the PDB file (2I0E). Carbon (yellow), nitrogen (blue) in BIS compounds are shown as spheres. The key residue for the substrate recognition, D470, is highlighted in pink. (**B**) CHO cells expressing CKAR were stimulated with 3 µM oxotremorine-M (oxo-M) at t = 0. Fluorescent signals from cells from the CFP channel are shown (far left). Oxo-M application induces phosphorylation of CKAR and a change in the CFP/YFP fluorescent ratio of the CKAR probe, shown as pseudocolor images. Images from indicated times are shown. (**C**) Cellular PKC activities in the presence (blue) or absence (white) of 200 nM BIS I. The black box indicates the presence of oxo-M. (**D**) Cellular PKC activity in the presence (orange) or absence (white) of 1 µM BIS IV. (**E**) Relative PKC activity in the presence of BIS I (blue) or BIS IV (orange) normalized to the PKC activity without inhibitors (white) derived from (C) and (D).

Bisindolylmaleimide I (BIS I) and bisindolylmaleimide IV (BIS IV) are structurally very similar PKC inhibitors (Fig. 1A). However, a crystal structure solved by others [11] and our molecular model show that BIS I interacts with the key substrate recognition residue, D470 [16], while BIS IV fits into the ATP binding pocket without occupying the substrate recognition groove (Fig. 1A). To examine the functional consequences for this difference, we measured cellular PKC activity using the cytoplasmic version of C kinase activity reporter, (CKAR), a fluorescence resonance energy transfer (FRET) based fluorescent probe [17]. CKAR was expressed in Chinese hamster ovary cells stably expressing the human m_1_ muscarinic acetylcholine receptor, CHO hm1 cells [8]. Upon application of 3 µM oxotremorine-M (oxo-M), CHO hm1 cells expressing CKAR showed a PKC response that reached its maximal activation within 20 sec (Fig. 1B). Preincubation with 200 nM BIS I or 1 µM BIS IV suppressed cellular PKC activities to a similar extent (BIS I 43.9±3.5% vs. BIS IV 57.4±3.5% of the control) (Fig. 1C and D). A higher potency of BIS I was consistent with the described higher affinity of BIS I than BIS IV [18]. When we compared the time courses of PKC activities with or without BIS compounds, we realized that the PKC responses from both BIS I and BIS IV treated cells were distorted rather than a miniature of the control responses. To further analyze this kinetic change, we compared relative PKC activities for BIS I and BIS IV treated cells (Fig. 1E). Relative PKC activities showed that BIS I gradually gained in potency, as indicated by a higher PKC activity at 6 sec than at 60 sec after activation (58.9±4.5% vs. 45.1±3.1% of the control, p<0.001). This change in the presence of BIS I was best fit with an exponential decay with a time constant (τ) of 8.2±0.3 sec. On the other hand, BIS IV gradually lost its potency; relative PKC activity was lower at 6 sec (35.5±2.5%) than at 60 sec after stimulation (58.3±3.2%, p<0.001). This increase in PKC activity was best fit with a single exponential association with τ of 25.5±1.3 sec.

### Activation states and potencies for BIS compounds

**Figure 2:**
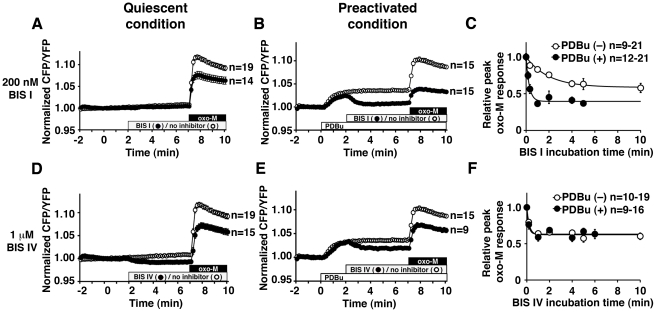
Activation status of PKC changes the inhibition kinetics of BIS I but not BIS IV. (**A**) Oxo-M responses with (closed circles) or without (open circles) BIS I in the quiescent condition. The boxes indicate the presence of 200 nM BIS I (grey) and 3 µM oxo-M (black). (**B**) Oxo-M responses with (closed circles) or without (open circles) BIS I in the preactivated condition. Cells were treated sequentially with 100 nM phorbol 12,13-dibutyrate (PDBu) (open box), 200 nM BIS I (grey box), and 3 µM oxo-M (black box). (**C**) Relative peak oxo-M responses from experiments similar to (A) and (B) with various incubation times with BIS I. Peak oxo-M responses under various BIS I incubation times are normalized to the peak response without BIS I treatment (t = 0). For instance, (A) represents two data points for the PDBu(–) condition, t = 0 min (no inhibitor, n = 19) and t = 5 min (BIS I, n = 14). (B) represents two data points for the PDBu(+) condition, t = 0 (no inhibitor, n = 15) and t = 5 min (BIS I, n = 15). Preactivation with PDBu (closed circles) facilitates PKC inhibition by BIS I compared to no PDBu (open circles). (**D**) Oxo-M responses with (closed circles) or without (open circles) 1 µM BIS IV in the quiescent condition. The control is the same as in (A). (**E**) Oxo-M responses with (closed circles) or without (open circles) 1 µM BIS IV in the preactivated condition. The control is the same as in (B). (**F**) Time course of relative peak oxo-M responses showing inhibition kinetics of BIS IV for preactivated PKC (closed circles) and the control (open circles) from experiments similar to (D) and (E) with various incubation times with BIS IV. Relative peak oxo-M responses with various incubation time with BIS IV are calculated using response at t = 0 as a reference. Panel (D) represents two data points for the PDBu(–) condition, t = 0 (no inhibitor, n = 19) and t = 5 min (BIS IV, n = 15). Panel (E) represents two data points for the PDBu(+) condition, t = 0 (no inhibitor, n = 15), and t = 5 min (BIS IV, n = 9).

To further examine whether the activation status of PKC changes BIS binding, we compared sensitivity to BIS I between preactivated PKC and quiescent PKC (Fig. 2A, B and C). Preactivation of PKC with 100 nM phorbol 12,13-dibutyrate (PDBu) promoted PKC inhibition by 200 nM BIS I (preactivated condition, 36.8±2.5% vs. quiescent condition, 63.0±7.5% of the control responses, p<0.01) (Fig. 2A and B). To clarify the mechanism for this facilitation, we measured inhibition kinetics for the two conditions by varying the incubation times with BIS compounds with or without preactivation. In the preactivated condition, BIS I reached its maximal inhibition within 60 sec incubation (Fig. 2C). This change was best fit with a single exponential decay with τ of 16.4±2.6 sec. In contrast, when BIS I was added in the quiescent condition, it required more than 4 min to reach the plateau inhibition, τ = 120.6±22.1 sec. On the other hand, 1 µM BIS IV inhibited oxo-M responses equally in both quiescent and preactivated conditions (Fig. 2D and E). Interestingly, BIS IV suppressed PKC within 1 min in both conditions, although the plateau inhibition was less than that of BIS I (Fig. 2F). In other words, neither inhibition kinetics nor potency of BIS IV were changed by preactivation of PKC (preactivation, τ = 12.9±6.4 sec vs. control, τ = 13.8±4.7 sec).

### The pseudosubstrate domain and BIS binding

**Figure 3:**
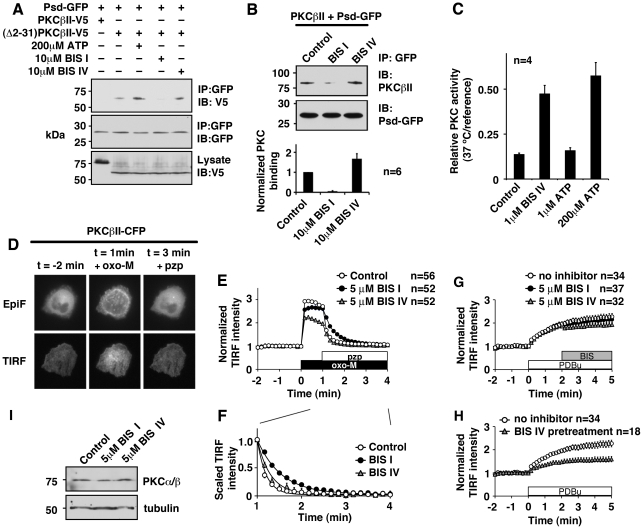
BIS compounds change how the pseudosubstrate domain interacts with PKC. (**A**) Immunoprecipitation from cell lysates expressing the pseudosubstrate domain fused with GFP (Psd-GFP) and V5 tagged wild-type PKCβII or the pseudosubstrate domain deleted PKCβII, (Δ2-31)PKCβII. The binding conditions are indicated. (**B**) *In vitro* binding between Psd-GFP and purified PKCβII in the presence of PDBu. Lower panel shows quantification from independent experiments. (**C**) Thermal stability of purified PKCβII in indicated conditions. Four independent experiments of triplicates are summarized. (**D**) Subcellular localization of CFP tagged PKCβII, PKCβII-CFP. Epifluorescent (EpiF) and TIRF (TIRF) images were collected sequentially from the same cell. Focal application of 300 nM oxo-M induced translocation of PKCβII-CFP, which was terminated by 100 µM pirenzepine (pzp). (**E**) Quantification of PKCβII-CFP localization by TIRF intensity from control (open circles), 5 µM BIS I (closed circles) or 5 µM BIS IV (grey triangles) treated cells. Application of oxo-M (black box) and pirenzepine (pzp, open box) are indicated. (**F**) Scaled time courses from (E) showing the deactivation process after application of pirenzepine. (**G**) Effects of BIS compounds on membrane location of activated PKCβII-CFP. The boxes indicate the presence of 100 nM PDBu (white) and BIS compounds (grey). No inhibitor (open circles), 5 µM BIS I (closed circles), 5 µM BIS IV (grey triangles) are indicated. (**H**) PDBu induced translocation of 5 µM BIS IV pretreated cells (grey triangles). No inhibitor control (open circles) is the same as in panel (G). (**I**) Immunoblots of PKCα/β and tubulin after two-hour treatments with 5 µM BIS I and 5 µM BIS IV. Tubulin is shown as a loading control.

If the pseudosubstrate domain interferes with the PKC-BIS I binding, then BIS I should interfere with the binding of the pseudosubstrate domain to the substrate recognition site. To test this, we fused the pseudosubstrate domain with GFP, Psd-GFP, and examined its binding to V5 tagged PKCβII, PKCβII-V5, by immunoprecipitation. Since synthesized pseudosubstrate peptides have been used as PKC inhibitors [19], Psd-GFP should replace the endogenous pseudosubstrate domain at the substrate recognition site in PKCβII. However, we could not detect PKCβII in Psd-GFP precipitates (Fig. 3A). We suspected that, in the non-activated condition, the intramolecular pseudosubstrate domain competes out Psd-GFP. Thus, we tested Psd-GFP binding with V5 tagged PKCβII lacking the pseudosubstrate domain, (Δ2-31)PKCβII. As expected, (Δ2-31)PKCβII coprecipitated with Psd-GFP in the non-activated condition. Addition of ATP further enhanced association between Psd-GFP and (Δ2-31)PKCβII, which is consistent with the evidence that ATP is a natural stabilizer for the pseudosubstrate-kinase interaction in some kinases [14], [20], [21], [22]. When Psd-GFP immunoprecipitation was performed in the presence of BIS I, coprecipitated PKCβII was greatly reduced (Fig. 3A). In contrast, addition of BIS IV promoted the association to an extent similar to that observed in the presence of ATP.

Since the above experiments used a deletion mutant, we next intended to confirm these effects of BIS compounds using wild-type PKCβII. Since our immunoprecipitation in the non-activated condition did not show stable association between PKCβII and Psd-GFP, we tested the interaction in the presence of a PKC activator. Activation of PKC should remove the intramolecular pseudosubstrate domain from its catalytic site, which would allow Psd-GFP to bind PKC. To this end, purified PKCβII was first treated with PDBu together with BIS I or BIS IV, then Psd-GFP bound resin was added to test PKC binding. In this preactivated condition, we could detect PKCβII in Psd-GFP precipitate as expected (Fig. 3B). When BIS I was included in the binding buffer, Psd-GFP could not bind PKCβII. In contrast, BIS IV promoted the association of Psd-GFP and PKCβII. In both series of experiments using (Δ2-31)PKCβII or wild-type PKCβII, BIS I interfered with binding of Psd-GFP to PKC. In contrast, BIS IV facilitated binding of Psd-GFP to PKC. This facilitation of the pseudosubstrate-catalytic site association by BIS IV is consistent with the fact that BIS IV is an uncompetitive inhibitor with respect to substrate peptides, which stabilizes the substrate peptide-PKC complex. These results also suggest that BIS IV promotes interaction between the pseudosubstrate and the catalytic site, which may increase the energy required for displacement of the pseudosubstrate from the catalytic cleft. To test this, we performed a thermal stability assay [23]. PKC activities were measured after incubation at 37°C for 30 min and compared with reference samples kept on ice. Wild-type PKCβII showed only 13.7±0.7% (n = 4) of PKC activity after a 37°C incubation (Fig. 3C). Inclusion of 1 µM BIS IV during the incubation improved PKC stability. ATP showed similar stabilization in a higher concentration (Fig. 3C).

These results suggest that BIS compounds modify the interaction between the pseudosubstrate and the catalytic site in distinct ways: BIS I repels the pseudosubstrate domain from the catalytic site, while BIS IV stabilizes the association of the pseudosubstrate domain and the catalytic site. Our new results suggest that BIS compounds can stabilize PKC either in the activated state or in the quiescent state depending on their molecular structures. The next question is whether such stabilizations occur within the cellular environment.

### BIS compounds and PKC translocation

To address this question, we measured the activation and deactivation processes of PKC using CFP tagged PKCβII, PKC-CFP. Conventional PKC is a unique kinase in that its conformational changes can be detected as a change in subcellular localization [15]. In the quiescent condition, conventional PKC localizes in the cytoplasm. Upon activation, PKC translocates to the plasma membrane. We utilized this unique feature of conventional PKC. To observe both the activation and the deactivation processes, we first stimulated cells with oxo-M for 1 min and then added a muscarinic receptor antagonist, pirenzepine, to terminate stimulation. We lowered the concentration of oxo-M to eliminate latency in the recovery process of PKC after adding pirenzepine. When PKC-CFP was expressed in CHO hm1 cells, PKC-CFP localized in the cytoplasm in the resting condition as expected (Fig. 3D). Application of oxo-M induced translocation of PKC-CFP to the plasma membrane. This translocation was clearly visible by epi-fluorescent microscopy, but quantification of this translocation was difficult (Fig. 3D). Total internal reflection fluorescent (TIRF) microscopy helped overcome this problem since translocation is directly converted into intensity of TIRF signals (Fig. 3D) [24]. In this setting, application of oxo-M induced a 2.9±0.1-fold increase in the TIRF signal at the 10 sec time point (Fig. 3E). Translocation was suppressed by 5 µM BIS IV throughout oxo-M application (p<0.0001), which supports our hypothesis that BIS IV stabilizes the quiescent state of PKC. For instance, the 10 sec time point showed a 2.2±0.1-fold increase with BIS IV. In contrast, treating cells with 5 µM BIS I had minor effects on the activation process; 2.6±0.1-fold increase (p<0.01) at the 10 sec point, which became indistinguishable from the control after 30 sec. These changes in translocation were not derived from changes in amount of PKC protein due to BIS treatments since immunoblots verified equivalent PKC amounts after 2 hr of BIS treatments (Fig. 3I): 126±16% (n =  3) for BIS I, 121±18% (n  =  3) for BIS IV, respectively.

On the other hand, BIS I did affect the deactivation process. In the control condition, PKC-CFP restored its cytoplasmic localization after application of 100 µM pirenzepine with τ of 12.1±0.6 sec (Fig. 3F). In the presence of BIS I, however, the deactivation process was delayed (τ = 32.3±0.7 sec, p<0.0001). Treatment with BIS IV had little effect on the deactivation process, τ = 17.7±1.1 sec (p>0.05). These results suggest that BIS IV can stabilize PKC in the quiescent conformation. We wondered whether BIS IV could switch activated PKC to the quiescent conformation in the presence of PDBu. To address this, we first activated PKC-CFP by PDBu and then added BIS compounds to see whether or not they affect membrane location of PKC-CFP. Application of PDBu induced slower translocation of PKC-CFP than oxo-M induced translocation, which is consistent with the slower activation of kinase activity (Fig. 2B). After 2 min incubation with PDBu, 5 µM BIS I or 5 µM BIS IV was applied (Fig. 3G). BIS I did not affect the membrane localization of activated PKC-CFP; 7.2±9.7% reduction compared to no inhibitor control at t = 3 min (p>0.05). BIV IV induced a small reduction of membrane PKC-CFP, 23.2±6.4% reduction when compared to no inhibitor control (t = 3 min, p<0.01). However, this reduction would be better attributed to prevention of further activation of PKC since the TIRF signal remained unchanged after application of BIS IV (t = 2 min vs. t = 3 min, p>0.05). We further tested how pretreatment with BIS IV changes PDBu induced PKC-CFP translocation. Similar to oxo-M induced translocation, pretreatment with BIS IV showed suppressed PDBu induced translocation (Fig. 3H, 51.0±9.5% of the control at t = 3 min). These results suggest that, even though BIS IV stabilizes PKC in the quiescent state, BIS IV binding *per se* is not sufficient to turn activated PKC into the quiescent state in the presence of PDBu. Taken together, these results suggest that BIS compounds can stabilize PKC in either the quiescent state or the activated state in living cells depending on how the compounds interact with the pseudosubstrate domain in the cellular environment.

### State-dependent inhibition of staurosporine and its derivative

**Figure 4:**
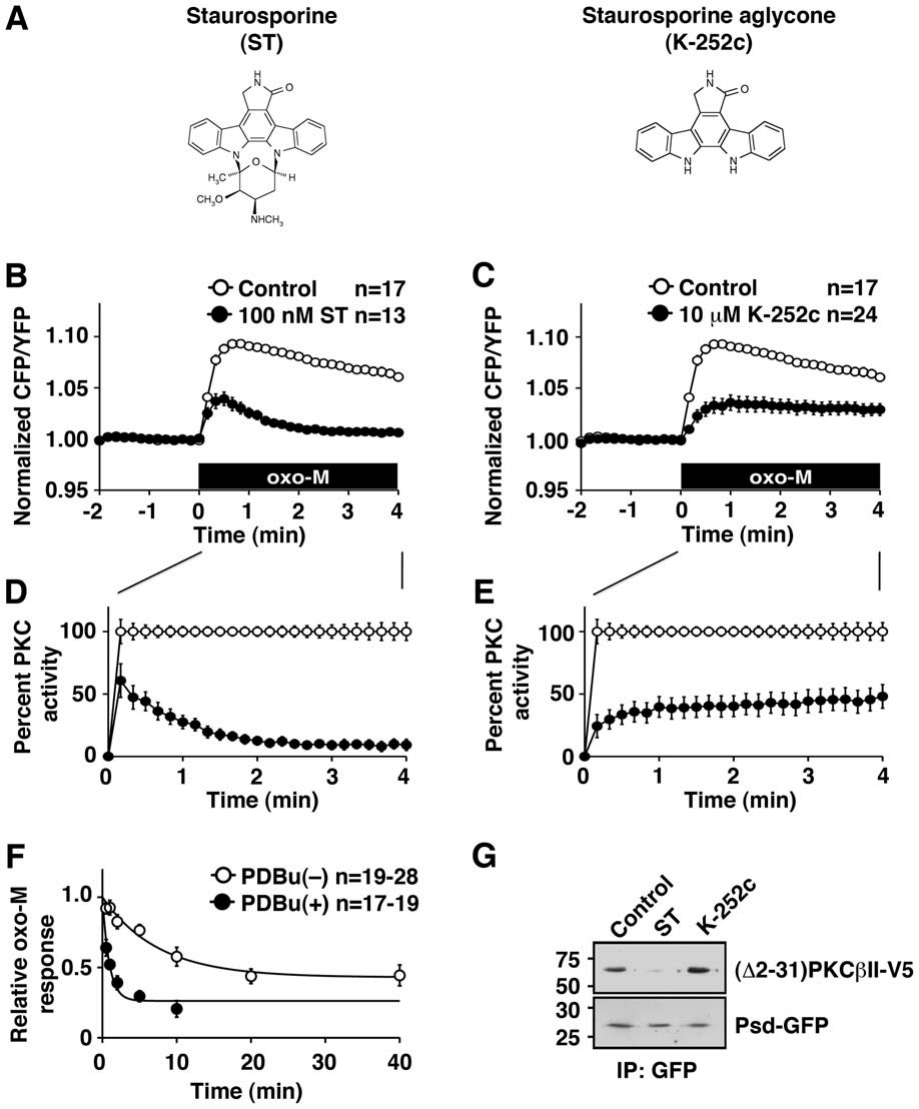
State-dependent inhibition by staurosporine and K-252c. (**A**) The molecular structures for staurosporine (left) and staurosporine aglycone, K-252c (right). (**B**) Cellular PKC responses with (closed circles) or without (open circles) 100 nM staurosporine (ST). Application of 3 µM oxo-M (black box) is indicated. (**C**) Cellular PKC responses with (closed circles) or without (open circles) 10 µM K-252c. (**D, E**) Scaled PKC activities from (B) and (C). (**F**) Inhibition kinetics measured as oxo-M responses of PKC incubated with 100 nM staurosporine for the indicated times with (closed circles) or without (open circles) 100 nM PDBu. (**G**) Coimmunoprecipitation of Psd-GFP and (Δ2-31)PKCβII in the control or in the presence of 10 µM staurosporine or 10 µM K-252c.

To test whether these effects are specific to BIS compounds or represent common phenomena for ATP competitive inhibitors, we performed a similar series of experiments using staurosporine and K-252c. Staurosporine is a prototypical molecule for ATP competitors and is a wide spectrum kinase inhibitor that shows high affinity binding to most kinases. K-252c is a staurosporine aglycone that lacks the amino group; the structural relation between K-252c and staurosporine is analogous to that of BIS IV and BIS I (Fig. 4A). Staurosporine treated cells showed a time-dependent increase in potency after activation of PKC by oxo-M, which was similar to but more prominent than for BIS I treated cells (Fig. 4B and D). For K-252c, it required 10 µM to achieve PKC inhibition similar to 100 nM staurosporine, which supports that the amino group in staurosporine provides additional interaction contributing to its high affinity (Fig. 4C). Even though 100 nM staurosporine suppressed almost all PKC activity at 4 min after oxo-M application, it suppressed less than 50% at 10 sec after activation (Fig. 4D). This decrease in PKC activity followed a single exponential decay with τ of 48.5±2.1 sec. K-252c treated cells, on the other hand, showed a time-dependent loss of potency, τ = 63.8±10.9 sec (Fig. 4E). The binding of staurosporine was facilitated by preactivation of PKC by PDBu (Fig. 4F). We attempted to perform similar time course experiments using K-252c. However, K-252c required more than 30 min incubation to show inhibitory effects, which suggests that cell permeability is the rate-limiting step for this compound, thus we could not assess inhibition kinetics of K-252c. On the other hand, immunoprecipitation experiments showed that staurosporine prevented association between Psd-GFP and (Δ2-31)PKCβII, while K-252c promoted the association (Fig. 4G). These results duplicated what was observed with the BIS compounds and suggest that state-dependent inhibitions are common phenomena for ATP competitive inhibitors.

## Discussion

In the present study, we have characterized the cellular pharmacology of several ATP competitive PKC inhibitors. Unlike conventional kinase assays that measure stationary activities, FRET based live-cell imaging analyses [25] enable us to measure real time PKC activities, which makes it optimal for analyzing the kinetics of kinase activation and kinase inhibition. By employing this technique, we identified that common PKC inhibitors are state-dependent inhibitors, which target either quiescent or activated PKC. This conclusion was derived from the following three supportive observations.

Firstly, these PKC inhibitors showed time-dependent changes in their potencies after activation of PKC. The time-dependent changes for both BIS I and BIS IV were best fitted by single exponential functions (Fig. 1), which suggests a single step transition to a new equilibrium. Interestingly, even though BIS I and BIS IV are structurally very similar to each other, the changes in potency after activation of PKC were opposite; BIS I showed an increase in potency while BIS IV exhibited a decrease in potency. These results suggest that BIS compounds have distinct affinities for either quiescent or activated PKC.

Secondly, BIS I preferentially inhibited preactivated PKC. This is evidenced by higher susceptibility to inhibition of preactivated PKC and a much faster time course to reach the plateau inhibition in preactivated PKC (Fig. 2). In contrast, BIS IV did not show preference for activated PKC. The key structural difference between BIS I and BIS IV is the amino group of BIS I that occupies the substrate recognition site of PKC (Fig. 1) [11], [18]. We have previously shown that BIS I is a competitive inhibitor not only for ATP but also for the substrate peptides [8]. Hence, competition between BIS I and the pseudosubstrate domain was suspected for the mechanism behind the preference for activated PKC of BIS I. Namely, the pseudosubstrate domain protects the substrate site from BIS I in quiescent PKC since the pseudosubstrate domain occupies the substrate recognition site in the quiescent state. This protective effect of the pseudosubstrate domain in the quiescent state is consistent with the slower inhibition kinetics of BIS I observed in the quiescent condition compared to the preactivated condition (Fig. 2C). In contrast, BIS IV did not show such facilitation of either potency or kinetics by preactivation of PKC. However, the time constants of BIS IV inhibition in both conditions were similar to that of BIS I in the preactivated condition, which suggests interference with BIS I inhibition in the quiescent PKC rather than facilitation in the preactivated PKC. Accordingly, our binding studies showed that BIS I bound PKC was unable to bind the pseudosubstrate domain (Fig. 3A and B). Collectively, these experiments suggest that the pseudosubstrate domain bound PKC allows limited access for BIS I, and is thus resistant to BIS I. On the other hand, BIS IV binding did not interfere with the pseudosubstrate domain of PKC, rather it promotes the binding. This is consistent with our previous observation that BIS IV is an uncompetitive inhibitor with respect to substrate peptides [8]. This mechanism indicates that BIS IV stabilizes the interaction between the pseudosubstrate domain and the catalytic site. Accordingly, our binding study and thermal stability assays showed that BIS IV stabilized the interaction between PKC and the pseudosubstrate domain (Fig. 3A, B and C). ATP has been known to stabilize the pseudosubstrate binding to the catalytic site [14]. Our thermal stability assay confirmed the stabilization effect of ATP as well as BIS IV. Since BIS IV has a higher affinity to PKC than ATP, BIS IV should have a higher Gibbs free energy for its binding. We speculate that this higher binding energy is an underlying mechanism for the suppression of cellular translocation of PKC in the presence of BIS IV; the stabilization effect of BIS IV exceeds that of the endogenous stabilizer, ATP.

Finally, BIS I bound PKC is stabilized in the activated conformation. This is suggested by a delayed recovery of cytosolic localization of PKCβII-CFP after termination of the activation signal (Fig. 3E and F). We previously demonstrated by *in vitro* experiments that BIS I stabilizes PKC in the activated conformation [8]. In the present study, we observed that such stabilization occurred in a cellular environment. This stabilization of the activated conformation is expected from our hypothesis since BIS I at the catalytic site prevents restoring of the interaction between the pseudosubstrate domain and the catalytic site (i.e. the quiescent state).

**Figure 5:**
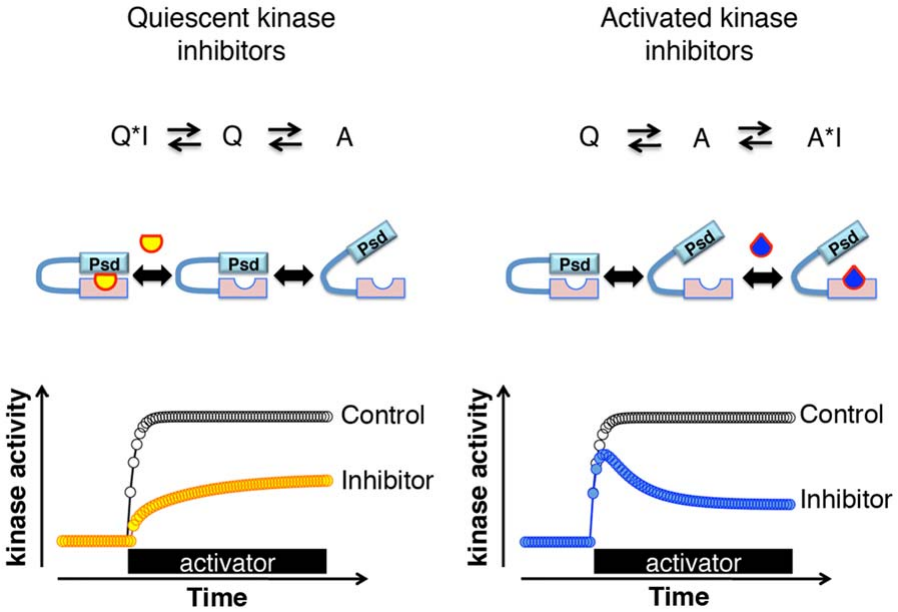
Models for state-dependent inhibition and its effects on kinase activities. Schematic diagrams (top), cartoons for relevant states of PKC (middle) and time course of PKC activities (bottom) for two types of state-dependent PKC inhibitor models. The diagram indicates inhibitor (I) and PKC in the quiescent state (Q) and activated state (A). The cartoons show PKC with the pseudosubstrate domain (Psd) and the catalytic domain for the corresponding states described above. Two types of inhibitors are indicated (yellow particle, blue particle). The trace with yellow circles at the bottom shows a conceptual model of slow activation of kinases in the presence of quiescent kinase inhibitors since the inhibitors stabilize PKC in the quiescent state. In contrast, activated PKC inhibitors bind PKC in the activated state and interfere with the deactivation process. The time lag between PKC activation and the inhibitor binding generates the transient activation of PKC (blue circles).

Taken together, we speculate that the binding of BIS I and the pseudosubstrate domain to the catalytic site are mutually exclusive (Fig. 5). If the pseudosubstrate domain binds the catalytic site, it cannot bind BIS I, which results in BIS I resistance. In contrast, if BIS I binds to the catalytic site, the pseudosubstrate domain cannot bind to the catalytic site, which stabilizes PKC in the activated state. We believe that the delayed deactivation of PKC (Fig. 3) induced by BIS I is equivalent to the "foot-in-the-door" effect described in state-dependent channel inhibitors [26], [27]. In short, BIS I targets the activated PKC.

On the other hand, BIS IV is an uncompetitive inhibitor with respect to the substrate peptide [8]. We speculate that BIS IV stabilizes the interaction between the pseudosubstrate domain and the substrate recognition site (Fig. 5). Our thermal stability assay showed that PKC was stabilized by BIS IV (Fig. 3C). Furthermore, translocation experiments showed that BIS IV treated cells had a reduced pool of PKC that could be activated, which also supports the hypothesis that BIS IV stabilizes PKC in the quiescent conformation. However, it is interesting that BIS IV treated cells did not show slower translocation, as might have been expected from the slower kinase activation (Fig. 1E). One possible explanation would be that the quiescent state supports the binding of BIS IV, and that the conformation of the kinase domain induced by inhibitor binding affects its binding to interaction partners such as cytosolic calcium, which impairs its translocation.

When we consider the gradual loss of BIS IV potency after activation (Fig. 1), we believe that the pseudosubstrate-BIS IV-catalytic site association reciprocally stabilizes their interaction. Thus, once PKC is activated, activation would break the pseudosubstrate-BIS IV interaction to lower BIS IV affinity, which would result in gradual loss of its potency. Taken together, we speculate that BIS IV counteracts the conformational change that dissociates the pseudosubstrate domain from the catalytic site, which suppresses the translocation of PKC.

Recently, a crystal structure of full-length PKCβII has been solved [12]. The study suggests a two-step activation process; disengagement of the C1A from the catalytic domain, which removes the pseudosubstrate domain from the catalytic site, followed by unclamping of the C1B site, which induces an allosteric change in the C-terminal NFD motif. Interestingly, the identified crystal structure was formed without diacylglycerol, but it did not show electron density for the pseudosubstrate domain [12]. We wonder if BIS IV or K-252c could assist in solving the structure of PKCβII in the quiescent conformation.

Despite the importance of state-dependent inhibition, not much attention has been paid to this aspect for kinase inhibitors. Real time monitoring of cellular kinase activity helped us to explore state-dependent inhibition. The fact that these state-dependent inhibitions were also observed for staurosporine, a wide spectrum kinase inhibitor, suggests that state-dependent inhibition is a common feature for ATP competitive inhibitors. In addition, we wish to emphasize that, as a consequence of state-dependent inhibition, kinase activity in the presence of kinase inhibitors is not a proportional miniature of the control response (Fig. 5). This feature is especially important for activated kinase inhibitors since transient activation remains in the presence of this type of inhibitor. For an example, if a pathway consists of a cascade of reactions in such a way that phosphorylation is only required as its trigger, then such pathway would not be fully inhibited by activated PKC inhibitors. Namely, the transient PKC activity in the presence of activated PKC inhibitors would be sufficient to activate the pathway. This limited efficacy of active PKC inhibitors due to the lag time of inhibitor binding could be an alternative mechanism for resistance to kinase inhibitors in addition to protection through scaffold proteins [8]. On the other hand, activated PKC inhibition would be beneficial for therapeutic purposes. Many pathogenic pathways involve constitutively activated kinases, while normal pathways remain quiescent until they are activated by physiological stimuli. Thus, activated kinase inhibitors would selectively target such pathological pathways. These state-dependent inhibitions would be a useful strategy to target selective conditions in signaling cascades.

## Materials and Methods

### Expression plasmids

CKAR expression plasmid was a generous gift from Dr. Alexandra Newton at UCSD [25]. We used the cytosolic version of CKAR cDNA, which has been described previously [17]. Psd-GFP was generated as follows: the oligo DNA encoding the pseudosubstrate domain of PKCβII (forward, 5′-GATCCCGCTTCGCCCGCAAAGGCGCCCTCCGGCAGAAGAACGTG-3′; reverse, 5′-AATTCACGTTCTTCTGCCGGAGGGCGCCTTTGCGGGCGAAGCGG-3′) was synthesized, annealed and ligated into pEGFP-C1 plasmid at BglII and EcoRI sites. DNA sequencing and immunoblot verified Psd-GFP constructs. V5 tagged PKCβII and (Δ2-31)PKCβII were made by PCR using wild-type PKCβII [28] and the following primers (full-length PKC forward, 5′- CACCATGGCTGACCCGGCTGCGGGGCCGCCGCCG-3′, (Δ2-31) forward, 5′-CACCATGCATGAGGTCAAGAACCACAAATTCACCGCCCGCTTCTTCAAGCAGCCC-3′, reverse, 5′-GCTCTTGACTTCGGGTTTTAAAAATTCAGAGTTAAC-3′). PCR fragments were subcloned using the pcDNA3.1 directional TOPO kit (Invitrogen, Carlsbad, CA). All PCR derived fragments were confirmed by sequencing.

### PKC inhibitors

Bisindolylmaleimide I, bisindolylmaleimide IV, staurosporine, K-252c and phorbol 12,13-dibutyrate were purchased from Sigma-Aldrich (St. Louis, MO). These compounds were dissolved in DMSO as stock solutions and diluted at least 1∶10,000 for actual experiments. For the experiments involving steady state inhibitions, cells were incubated with relevant inhibitors for between 20 min and 2 hours.

### Cell culture and transfection

CHO cells stably expressing human m_1_ muscarinic receptor [29] were cultured in alpha minimum essential medium, 5% fetal bovine serum, 500 µg/ml G418 at 37°C in 5% CO_2_. HEK 293A cells (Invitrogen, Carlsbad, CA) were cultured in Dulbecco's Modified Eagle's Medium containing 10% fetal bovine serum. For transfections, cells were grown to 30% confluence and transfected (2 µg total plasmid DNA and 8 µl Trans-IT1 per 10 cm dish) according to the manufacturer's instruction (Mirus Bio, Madison, WI).

### Live-cell imaging

Fluorescence emission was acquired using an inverted microscope IX-81 (Olympus, Tokyo, Japan) and ImageEM CCD camera (Hamamatsu photonics, Shizuoka, Japan) controlled by MetaMorph 7.6.3 (Molecular Devices, Sunnyvale, CA). The excitation light was generated by Lambda LS (Sutter Instruments, Novato, CA) and passed through a S436/10x or S500/20x filter. The light intensity was reduced to 3% for 436 nm and 0.07% for 500 nm to minimize photobleaching. Dual-emission images were obtained simultaneously through a dual-view module (Photometrics, Tucson, AZ) with ET535/30m, ET480/40m emission filters and a T505lpxr dichroic mirror (Chroma Technology, Bellows Falls, VT). Exposure time was 100 ms; images were taken every 1.5 s for experiments shown in Fig. 1 and 10 s for other experiments. For total internal reflection fluorescent (TIRF) experiments, a 445 nm diode laser (Coherent, Santa Clara, CA) with an acousto-optic tunable filter was used with a TIRF module (Olympus, Tokyo, Japan) equipped with IX-81. Illumination paths between epifluorescence and TIRF were switched manually for the sequential imaging shown in Fig. 3D. Fluorescent intensities from each cell were background subtracted and the CFP/YFP ratio was calculated. Since the basal CFP/YFP ratio varies between cells, basal values before stimulation by oxo-M were normalized and pooled for each experimental group.

Cells were cultured on round cover glasses (18 mm diameter) and washed twice before mounting onto the microscope with a solution consisting of 144 mM NaCl, 5 mM KCl, 2 mM CaCl_2_, 0.5 mM MgCl_2_, 10 mM glucose, 10 mM HEPES (pH 7.4). All data are presented as mean ± SEM.

### PKC Immunoprecipitation

Transfected HEK cells were lysed with Hypotonic buffer containing 10 mM NaCl, 1.5 mM MgCl_2_, 1 mM dithiothreitol, 5 mM EGTA, 10 mM HEPES (pH 7.4), 0.4% Nonidet P-40 and complete mini protease inhibitor cocktail (Roche Diagnostics, Indianapolis, IN). After centrifugation at 22,000 g for 20 min at 4°C, the lysate was precleared with protein G resin, and then immunoprecipitated with rabbit GFP antibody (Abcam, Cambridge, MA), and protein G resin. Relevant samples contained 200 µM ATP or 10 µM PKC inhibitors. Psd-GFP bound resin was washed three times with HSE buffer containing 150 mM NaCl, 5 mM EDTA, 5 mM EGTA, 20 mM HEPES (pH 7.4), 1% Triton X-100 and analyzed by SDS-PAGE followed by western blot.

### PKC binding assay

Psd-GFP was purified from transiently transfected HEK cells by immunoprecipitation. Transfected cells were lysed with HSE buffer and complete mini protease inhibitor cocktail. After centrifugation at 22,000 g for 15 min at 4°C, the lysate was precleared with protein G resin, and then immunoprecipitated with rabbit GFP antibody (Abcam, Cambridge, MA) and protein G resin. Psd-GFP bound resin was washed twice with HSE buffer followed by twice with HSE buffer containing 650 mM NaCl then twice with HSE buffer. For the binding assay, 60 ng PKCβII (EMD chemicals, Gibbstown, NJ) in 10 µl binding buffer was incubated 5 min at room temperature with or without 10 µM PKC inhibitors. Then, purified Psd-GFP attached to beads was added and further incubated 15 min at room temperature. After three washes with the binding buffer plus relevant PKC inhibitors, associated proteins were analyzed by western blots using a monoclonal antibody toward PKCβ (BD bioscience, San Jose, CA). The binding buffer consisted of 0.1% Triton X-100, 150 mM NaCl, 100 nM PDBu, 1 mM DTT, 10 mM HEPES (pH 7.4).

### PKC thermal stability assay

Aliquots of PKCβII (EMD chemicals, Gibbstown, NJ) were prepared and incubated for 30 min at 37°C in a buffer containing 1 mM CaCl_2_, 1 mM dithiothreitol, 1 mM sodium orthovanadate, 25 mM β-glycorol phosphate, 20 mM MOPS (pH 7.2). Relevant samples contained ATP and BIS IV. Reference samples were kept on ice during the incubation. PKC kinase activity was measured using ^32^P-ATP and PKC assay kit (Millipore, Billerica, MA). Experiments were done as triplicates.
